# Educational status and beliefs regarding non-communicable diseases among children in Ghana

**DOI:** 10.1186/s12889-018-5211-5

**Published:** 2018-03-05

**Authors:** Delali M. Badasu, Aaron A. Abuosi, Francis A. Adzei, John K. Anarfi, Alfred E. Yawson, Deborah A. Atobrah

**Affiliations:** 1 Regional Institute for Population Studies, University of Ghana, Legon, Accra Ghana; 2 Department of Public Administration and Health Services Management, University of Ghana Business School, Legon, Accra Ghana; 3 Department of Community Health, University of Ghana Medical School, Legon, Accra Ghana; 4 Institute of African Studies, University of Ghana, Legon, Accra Ghana

**Keywords:** Non-communicable diseases, Children, educational status, Belief, Ghana

## Abstract

**Background:**

Increasing prevalence of non-communicable diseases (NCDs) has been observed in Ghana as in other developing countries. Past research focused on NCDs among adults. Recent researches, however, provide evidence on NCDs among children in many countries, including Ghana. Beliefs about the cause of NCDs among children may be determined by the socioeconomic status of parents and care givers. This paper examines the relationship between educational status of parents and/or care givers of children with NCDs on admission and their beliefs regarding NCDs among children.

**Methods:**

A total of 225 parents and/or care givers of children with NCDS hospitalized in seven hospitals in three regions (Greater Accra, Ashanti and Volta) were selected for the study. Statistical techniques, including the chi-square and multinomial logistic regression, were used for the data analysis.

**Results:**

Educational status is a predictor of care giver’s belief about whether enemies can cause NCDs among children or not. This is the only belief with which all the educational categories have significant relationship. Also, post-secondary/polytechnic (*p*-value =0.029) and university (p-value = 0.009) levels of education are both predictors of care givers being undecided about the belief that NCDs among children can be caused by enemies, when background characteristics are controlled for. Significant relationship is found between only some educational categories regarding the other types of beliefs and NCDs among children. For example, those with Middle/Juniour Secondary School (JSS)/Juniour High School (JHS) education are significantly undecided about the belief that the sin of parents can cause NCDs among children.

**Conclusions:**

Education is more of a predictor of the belief that enemies can cause NCDs among children than the other types of beliefs. Some categories of ethnicity, residential status and age have significant relationship with the beliefs when background characteristics of the parents and/or care givers were controlled for.

## Background

The prevalence of non-communicable diseases (NCDs) which were formerly characteristic of developed countries, has become a major public health concern in developing countries also. Currently, more people suffer from NCDs in developing countries, including Ghana, than in developed areas. Three quarters (28 million) of the 38 million deaths from NCDs recorded each year now occur in these countries. Globally, NCDs are associated with the elderly, but evidence shows that children and adults are also having the diseases [1–3]. In Ghana, clinical manifestations of some NCDs, including diabetes, hypertension, cardiovascular diseases and metabolic syndromes associated with overweight and obesity, have been increasing in the adult population over the past two decades or so [4–6].

A major area of concern regarding NCDs in Ghana that requires examination by researchers is the explanation of the diseases or what causes them. Even though biomedical explanation and understanding of NCDs have been widespread in the country and patients seek modern health services for treatment, the traditional health system has also been a source of treatment, either solely or in combination with others. This situation has been described as pluralism in health behaviour [7, 8]. In contemporary Ghana, both traditional and scientific health systems do exist and are very different with respect to the explanation of the causes of diseases as well as the approach to their prevention and cure [9]. Moreover, Ghanaian society demonstrates strong adherence to religious beliefs regarding NCDs among both patients and care providers. The findings of a study on health service delivery at the Korle Bu Teaching Hospital, which examined “the role of religious beliefs in the delivery of care in the hospital setting” notes the deep rooted Christian belief in Ghana and how the whole nursing profession and the health delivery behavior of nurses has been motivated by religious convictions. The study pointed out that religious beliefs support the nurses’ capacity to cope with the resource constraints (such as extreme shortage of personnel and equipment) that characterize the health facility and even form the basis of how society excuses the nurses from sub-optimal performance [10, 11]. Religious beliefs about diseases can make parents of children with NCDs resign to fatalistic ideas about them. They may also influence the kind of health service parents and/or care givers access for the treatment of the NCD the child is suffering from.

Under the traditional medical system, the cause of disease is invariably linked to supernatural powers, and for that matter, magico-religious acts and concepts are used to cure diseases of all kinds. However, physical cures and treatment with herbs are also employed [9–12]. Conditions of malnutrition such as kwashiorkor (a protein-energy deficiency syndrome) and convulsion are attributed to spirits and supernatural forces [13, 14]. Consequently, explanation about some diseases suffered by people, including NCDs among children may be generally attributed to witchcraft, punishment and/or attacks from spiritual sources and ancestors [13, 15]. Stigmatization or discrimination against persons with NCDs also exists as is the case with other diseases, including Human Immunodeficiency Virus (HIV), mental illness and others that are perceived as “abnormal” or capable of causing harm to others or making the patient unfit for human society [16]. It may be expected that formal education can erode traditional beliefs about the causes of NCDs. But the pluralism in health-seeking behavior in Ghana suggests that the traditional explanations of NCDs and associated beliefs prevail among the educated.

In Ghana, traditional perceptions, beliefs and behaviours regarding NCDs prevent some parents from exposing their children in public or taking them to modern health facilities. Consequently, survival of children with NCDs depends on the beliefs of their parents and/or care givers, even when modern health facilities are available and affordable for treatment. The dependence of children on their parents and/or care givers for the treatment or management of non-communicable diseases, therefore, requires that researchers examine parents and/or caregivers’ beliefs regarding NCDs among children.

Also, studies on NCDs in most countries focus on adult populations. Indeed the risk factors associated with such diseases are largely lifestyle or behavioural. Genetic factors that are associated with NCDs have also been recognized. Thus, Ghana’s response to the epidemiological transition and management of these diseases targets nutrition behaviour and related areas, including drinking of adequate amount of water as well as exercise and rest [17, 18].

Recently, however, interest has been growing in research on NCDs among children for two major reasons. Firstly, the age at which NCDs, such as hypertension and diabetes set in has gradually reduced as evident in several studies. Overweight and obesity and elevated blood pressure have been among the most cited conditions among children in Ghana and in different parts of the world, including the United States of America, Canada and Europe [19–23]. Secondly, in the case of children in African countries in particular, including Ghana, the introduction of modern medical technology into the countries since the early parts of the Twentieth Century [24] has also contributed to the increasing survival rate of children who suffer from NCDs and congenital conditions. Research interest in them has been increasing.

This paper, therefore, examines beliefs of parents and/or care givers (relatives and non-relatives) regarding NCDs among children at Ghana’s two leading tertiary and teaching hospitals and other hospitals where such children were on admission. The general objective was to find out the influence of the formal educational status of parents and or care givers on beliefs regarding NCDs among children.

## Methods

### Study sites

The paper is based on a study conducted in three out of the ten regions of Ghana. The health facilities in which the interviews were conducted in the three regions include, Korle Bu Teaching Hospital (KBTH), the Princess Marie Louie Children’s Hospital in Accra (Greater Accra Region) and Komfo Anokye Teaching Hospital (KATH) in Kumasi (Ashanti Region). In the Volta Region, the interviews were conducted in four hospitals, including Ho Regional Hospital, Ho Municipal Hospital, Battor Catholic Hospital, and Mater Ecclesia to attain the minimum number of children required. The three regions were purposively selected in order to include all the major ethnic groups in the country, since beliefs about diseases is determined by cultural practices and beliefs. The two teaching hospitals are the leading referral hospitals for non-communicable diseases in Ghana. The Korle Bu Teaching Hospital also receives referred cases from the West African sub-region because it is the leading hospital in providing some specialist services and medical care in the sub-region.

The study was done at health facilities because it is difficult to conduct such a study in communities as stigma is attached to the NCDs. Parents and/or care givers would not like to participate in the study. However, in the health facilities on the other hand, they were willing to do so. Probably some reached the health facility before they were informed about the disease their children were suffering from.

### Sample size determination

The sample size was determined using OpenEpi, Version 3, open source calculator—SSPropor. It was based on the following equation:

$$ Sample\ size\ \boldsymbol{n}=\left[\mathbf{DEFF}\ast \mathbf{Np}\ \left(\mathbf{1}-\mathbf{p}\right)\right]/\left[\right({\mathbf{d}}^{\mathbf{2}}/{\mathbf{Z}}_{\mathbf{1}-\boldsymbol{\upalpha} /\mathbf{2}}^{\mathbf{2}}\ast \left(\mathbf{N}-\mathbf{1}\right)+\mathbf{p}\ast \left(\mathbf{1}-\mathbf{p}\right)\Big] $$

Where,

*n* = sample size

*DEFF* = design effect (used in cluster surveys)

*N* = population size

*P* = the hypothesized % frequency of outcome factor in the population

q = 1-p

*d* = confidence limits

Since the respondents were in-patients, it was expedient to determine the sample size from the population of in-patients with NCDs in the hospitals selected, but this was difficult to obtain. However, OpenEpi calculator permits a default population of 1,000,000 as the maximum population size to determine the largest sample size, and a default hypothesized percentage (%) frequency of outcome factor of 50% (http://www.openepi.com/SampleSize/SSPropor.htm). The hypothesized % frequency of outcome factor in the population, provides an educated guess of the percent of the population with the outcome of interest. In this study the outcome variables of interest were three: 1) the belief that NCDs among children can be caused by enemies; 2) the belief that NCDs among children can be caused by parent’s sin; and 3) the belief that NCDs among children can be caused by someone else. Since respondents were supposed to be contacted personally in the hospitals for interview, the study adopted 80% as the hypothesized frequency of patients responding to the questionnaire on the three outcome variables. With the hypothesized frequency of 80% and confidence limits as ±5, the confidence interval would be 80% ±5%, that is, (75%, 85%). Based on these specifications, the sample size generated by OpenEpi calculator for the study was 246. This was rounded off to 250. However, the actual respondents were 225, giving a response rate of 90%.

### Data collection

The interviews were conducted after consent forms were administered to the participants. The Institutional Review Board at the Noguchi Medical Research Centre, University of Ghana granted an ethical certificate for the study.

A quantitative research instrument was administered to participants who agreed to be interviewed. The questionnaire consisted of five modules, including socio-demographic background of the participants, knowledge of NCDs, beliefs about NCDs among children, health-seeking behavior of parents and/or care givers of the children and issues on tertiary health delivery policy regarding children in Ghana. The interviews were conducted between January and June 2013. A total of 225 parents and/or care givers, aged 18 years and above, were selected from the hospitals for the interview. None of the children on admission was interviewed at the health facility for this aspect of the study. The paper is based on the module on attitude and beliefs regarding NCDs among children aged 17 years and below. But only the questions on beliefs in the module were used for this paper.

### Data analysis

The independent variable is the level of education and the dependent variable is beliefs. Three questions were asked to capture the beliefs of parents in terms of what causes chronic diseases in children: 1) Can children’s NCDs be caused by enemies?; 2) Can the children’s NCDs be caused by parents’ sins?; and 3) Can someone else be responsible for the children’s chronic conditions? The responses for each of the three questions are: 0 = “No”, 1 = “Yes” 2 = “undecided”). A positive response to these questions is considered as accepting the belief or having the belief while a negative response is considered as not holding on to the belief. At the multivariate analysis level, those who responded “No” to each of the questions (do not hold on to the beliefs) were used as the reference category. The control variables are: age, sex, religion, place of residence, and ethnicity.

Frequency tables were used to present the background characteristics of the respondents. At the bivariate level, Chi-Square tests were used to examine the association between level of education of parents and/or care givers’ and beliefs regarding NCDs among the children. Furthermore, multinomial logistic regression was used to show the independent effect of parents and/or care givers’ level of education on the three beliefs, controlling for their background characteristics. The cut-off for statistical significance for the results was *p* = 0.05.

## Limitations of the study

The sample for the study was drawn solely from the health facilities that admit children suffering from non-communicable diseases. It is likely that the parents/care givers may be psychologically influenced by the health facility environment and provide answers to questions superficially to meet the expectations of the researchers or interviewers. In the communities, people’s views are supported by the collective views that are based on the social values and perspectives. There could be differences in the responses from these two settings. The health facility environment has the “natural” setting for the respondents, however, as they are experiencing the situation on which they are giving their views. Their responses are a result of their experience regarding the NCDs among their children, but not imaginations of other people’s situation. But those who do not accept the beliefs probably did so because they are in the hospital environment.

Generalizability of the results is not possible since the study did not include children in the community. At the community level, responses may be different from those at the health facility.

## Results and discussion

In all, 225 parents and/or care givers at seven health facilities in three regions of Ghana were interviewed. They were 169 (75.1%) females and 56 males (25%). In Ghana, females are primary care givers to children. They are supported by their kin to perform care tasks. The sex distribution of the parents and/or care givers at the health facilities is a reflection of what pertains in the wider Ghanaian society.

The other socio-demographic/economic characteristics of the participants are shown in Table 1. The vast majority of the participants have formal education, with the highest percentage (42.3%) having primary and middle/Junior Secondary School/Junior High School education. Almost a third (31.8%) have secondary or post-secondary education while close to a fifth (18.2%) have university education. Less than a tenth (7.7%) have no formal education. Compared to the total national population, the educational status of the participants is relatively high. Most (72.9 Percent) of the parents and/or care givers are 44 years and below. Almost 9 out of every 10 (86.6%) of the parents and/or care givers are Christians and the rest are affiliated to Islam (12%) and 1.4% traditional religion. Overall, 90.7% reside in urban areas, while 9.3 are rural dwellers. Most of them are of Akan (55.6%) and Ewe (17.6%) ethnic backgrounds, just as it is for the total national population.

**Table 1:** Background characteristics of the respondents

Background characteristics	Number	Percentage
Level of education		
No education	17	7.7
Primary	26	11.8
Middle/JHS	67	30.5
Secondary/SHS	41	18.6
Post-secondary	29	13.2
University	40	18.2
Age Group		
< 25	31	14.7
25–34	72	35.5
35–44	55	22.7
45–54	20	8.9
55–64	6	2.7
65+	4	1.8
Sex		3.3
Male	56	24.9
Female	169	75.1
Religion		
Catholic	18	8.7
Protestant/Anglican	76	36.5
Charismatic/Pentecostal	86	41.4
Traditional/spiritualist	3	1.4
Moslem	25	12.0
Place of residence		
Urban	185	90.7
Rural	19	9.3
Ethnic group		
Akan	125	55.6
Ga-Dagme	21	9.3
Ewe	40	17.8
Mole-Dagbani	17	7.6
Other Ghanaian	22	9.8

*N* = 220

Source: January–June 2013

The variation between the background characteristics of the parents and/or care givers and the belief that enemies can cause NCDs among children is presented in Table 2. The focus is on those who believe that their children’s NCDs could be caused by enemies. It can be observed from Table 2 that parents and/or care givers with no education reported the highest proportion of people (58.8%) who have the belief that their children’s NCDs could be caused by enemies while the least is recorded among those with post-secondary education (22.2%). Generally, the proportion having the belief that children’s NCDs could be caused by enemies is lower among those with higher levels of education. Also, the females (33.6%) recorded a little more than twice the percentage of males (16.3%) who believe that NCDs among children could be caused by enemies. In Ghana, females have a far lower educational status than males. Education erodes traditional beliefs about the causes of diseases, so parents and/or care givers with no education should be expected to report the highest proportion of those having that belief.

**Table 2:** Background characteristics and belief that enemies can cause NCDs on children

Characteristics	No	Yes	Undecided	Chi-Square	*P*-Values
Level of education					
No education	23.5	58.8	17.7	15.023	0.100
Primary	61.5	30.8	7.7		
Middle/JHS	64.2	26.8	9.0		
Secondary/SHS	65.7	28.6	5.7		
Post-secondary	66.7	22.2	11.1		
University	77.3	22.7	0.0		
Age Group					
< 20	37.5	25.0	37.5	32.432	0.070
20–24	66.7	33.3	0.0		
25–29	67.9	17.9	14.2		
30–34	59.3	37.0	3.7		
35–39	71.4	25.7	2.9		
40–44	56.7	36.7	6.6		
45–49	68.8	31.2	0.0		
50–54	45.5	36.4	18.1		
55–59	100.0	0.0	0.0		
60–64	25.0	50.0	25.0		
65–69	50.0	0.0	50.0		
Sex					
Male	76.7	16.3	7.0	5.277	0.071
Female	58.0	33.6	8.4		
Religion					
Catholic	69.2	30.8	0.0	6.115	0.634
Protestant/Anglican	65.6	26.6	7.8		
Charismatic/Pentecostal	56.8	33.8	9.4		
Traditional/spiritualist	33.3	33.3	33.4		
Moslem	71.4	19.1	9.5		
Place of residence					
Urban	69.0	22.6	8.4	12.306	0.002
Rural	37.5	62.5	0.0		
Ethnic group					
Akan	67.9	24.8	7.3	9.441	0.306
Ga-Dagme	52.4	42.9	4.7		
Ewe	56.0	40.0	4.0		
Mole-Dagbani	66.7	20.0	13.3		
Other Ghanaian	43.8	37.5	18.7		

Source: Field work January–June 2013

Regarding religious affiliation, about one-third of the parents and/or care givers who are Catholic (30.8%), Charismatic/Pentecostal (33.8%) and Traditionalist/spiritualist (33.3%) are of the opinion that children’s NCDs could be caused by enemies, whereas, less than 20% of the Moslems (19.1%) have this opinion. In Ghana, belief that non-communicable diseases are caused by spiritual forces, their agents or ancestors is held among Christians, particularly Pentecostal and Charismatic congregations, and this accounts for the patronage of prayer camps by sick persons seeking cure for diseases of all kinds. Further, a higher proportion of parents living in rural areas (62.5%), compared to urban residents, and a higher proportion of those who are Ga-Dagme (42.9%) and Ewe (40.0%) have this belief compared to other ethnic groups. A far lower percentage of parents and/or care givers at the health facilities with urban background (22.6%) compared to those with rural background (62.5%) believe that someone could NCDs among children. Half of the parents who are between the ages of 60–64 years old believe that children’s NCDs are caused by enemies and none of those who are 55–59 years and 65–69 years old has this belief.

Table 3 is on the belief that children’s NCDs could be caused by parents’ sins, by the background characteristics of the parents and/or care givers. The results show that parents and care givers with no education reported the highest percentage (29.4%) of those who believe that children’s NCDs is caused by parents’ sins, while those with Middle/JHS education reported the lowest percentage. A higher proportion (16.3%) of parents and/or care givers who are males compared to females (11.9%) have this belief. Those who are Catholics reported the highest proportion of people who believe that children’s NCD condition is due to parents’ sins. However, none of those in the Traditionalist/Spiritualist category has this belief, though such beliefs characterize that religion. This indicates that affiliation with a religion may not mean acceptance of all its practices and beliefs. Also, some Traditionalists/Spiritualists in contemporary Ghana are highly educated. They may accept biomedical and other explanations of diseases instead of the supernatural explanations that are associated with the Traditionalist/Spiritualist religion. This may also account for the relatively high percentage (33.3%) of those with Traditionalist/Spiritualist religion who were indecisive on their response to the statement. More than one in ten respodents in urban (11.0%) and rural areas (12.5%), believe that a parent’s sin can cause NCDs among children. The Ga-Dagme (23.8%) recorded the highest percentage of those who have this belief. Three-quarters (75.0%) of the parents and or care givers who are 60–64 years have this belief, while no parent and or care giver within the following age groups has this belief: 25–29 years, 55–59 years and 65–69 years.

**Table 3:** Background characteristics and belief that parents' sin can cause NCDs on children

Characteristics	No	Yes	Undecided	Chi-Square	*P*-values
Level of education					
No education	52.9	29.4	17.7	13.69	0.188
Primary	76.9	11.5	11.6		
Middle/JHS	86.6	9.0	4.4		
Secondary/SHS	77.1	17.1	5.7		
Post-secondary	83.3	11.1	5.6		
University	90.9	9.1	0.0		
Age Group					
< 20	87.5	12.5	0.0	39.609	0.012
20–24	72.2	16.7	11.1		
25–29	85.7	0.0	14.3		
30–34	92.6	7.4	0.0		
35–39	88.6	5.7	5.7		
40–44	66.7	26.7	6.6		
45–49	87.5	12.5	0.0		
50–54	63.6	18.2	18.2		
55–59	100.0	0.0	0.0		
60–64	25.0	75.0	0.0		
65–69	100.0	0.0	0.0		
Sex					
Male	81.4	16.3	2.3	1.974	0.373
Female	80.4	11.9	7.7		
Religion					
Catholic	76.9	23.1	0.0	7.593	0.474
Protestant/Anglican	84.4	9.4	6.2		
Charismatic/Pentecostal	75.7	16.2	8.1		
Traditional/spiritualist	66.7	0.0	33.3		
Moslem	85.7	9.5	4.8		
Place of residence					
Urban	82.6	11.0	6.4	0.889	0.641
Rural	75.0	12.5	12.5		
Ethnic group					
Akan	86.2	11.0	2.8	16.746	0.033
Ga-Dagme	66.7	23.8	9.5		
Ewe	68.0	12.0	20.0		
Mole-Dagbani	93.3	6.7	0.0		
Other Ghanaian	68.8	18.8	12.4		

Table 4 shows the variation between the background characteristics of the parents and/or care givers and the belief that someone else is responsible for NCDs among children. Close to two-thirds (62.5%) of those with no education believe that someone else is responsible for their child’s condition, while about one-third in the other education categories have this belief. A higher percentage of females (40.6%) than males (18.4%) and as high as two-thirds (66.7%) of those who have Traditionalist/Spiritualist religion had this belief. A higher proportion of parents in the rural areas (43.8%) than in urban areas (31.9%) have this belief. Among the ethnic groups, the highest percentage of those who have this belief are Ewe (42.0%). Further, half of the parents aged 60–64 and 65–69 years believe that someone else is responsible for their child’s condition, while none of those aged 55–59 has this belief. It is expected that older parents and/or care givers would hold on to the cultural practices that promote such beliefs.

**Table 4:** Background characteristics and belief that someone else is responsible NCDs among children

Characteristics	No	Yes	Undecided	Chi-Square	*P*-values
Level of education					
No education	25.0	62.5	12.5	8.439	0.586
Primary	52.0	36.0	12.0		
Middle/JHS	51.6	32.8	15.6		
Secondary/SHS	55.9	32.4	11.7		
Post-secondary	63.2	31.6	5.2		
University	58.8	35.3	5.9		
Age Group					
< 20	62.5	25.0	12.5	17.188	0.753
20–24	52.9	29.4	17.7		
25–29	56.0	28.0	16.0		
30–34	44.0	48.0	8.0		
35–39	44.1	44.1	11.8		
40–44	42.9	39.3	17.8		
45–49	66.7	33.3	0.0		
50–54	63.6	27.3	9.1		
55–59	100.0	0.0	0.0		
60–64	25.0	50.0	25.0		
65–69	50.0	50.0	0.0		
Sex					
Male	71.1	18.4	10.5	7.437	0.024
Female	47.1	40.6	12.3		
Religion					
Catholic	84.6	15.4	0.0	8.268	0.408
Protestant/Anglican	50.8	34.4	14.8		
Charismatic/Pentecostal	47.1	38.2	14.7		
Traditional/spiritualist	33.3	66.7	0.0		
Moslem	55.0	35.0	10.0		
Place of residence					
Urban	56.3	31.9	11.8	1.021	0.6
Rural	43.8	43.8	12.4		
Ethnic group					
Akan	50.0	37.3	12.7	2.001	0.981
Ga-Dagme	55.0	35.0	10.0		
Ewe	64.0	42.0	8.0		
Mole-Dagbani	46.2	38.5	15.3		
Other Ghanaian	50.0	37.5	12.5		

Source: Field work January–June 2013

### Level of education and the belief that enemies can cause disease in children

Table 5 shows the relationship between level of education and belief that NDCs can be caused by enemies. The results show that there is a significant relationship between level of education and having this belief. Compared with parents and/or care givers with no education, parents with Primary, Middle/JSS/JHS, Secondary/SHS, Post-Sec/Polytechnic and university education are less likely to believe that NCDs among children can be caused by enemies (75.0%, 79.1%, 78.3%, 83.3% and 85.3%, respectively). Generally, the likelihood of believing that children’s NCDs can be caused by enemies reduces with increase in the level of education. Moreover, those with primary and Middle/JSS/JHS education are significantly less likely (91.1% and 90% respectively) to be undecided about the belief that NCDs among children can be caused by enemies.

**Table 5:** Level of education and the belief that enemies can cause NCDs among children

Variable	Yes		Undecided about the belief	
	*P*-values	Odds ratio	*P*-values	Odd ratio
Education				
No education (RC)		1.000		1.000
Primary	0.047	0.250^**^	0.011	0.089^**^
Middle/JSS/JHS	0.011	0.209^**^	0.002	0.100^**^
Sec/SHS/Vocational	0.022	0.217^**^	0.051	0.248
Post-Sec/Polytechnic	0.024	0.167^**^	0.718	0.774
University	0.010	0.147^**^	0.680	0.756

The reference category is “No” (Does not accept the belief

Yes = Accepted belief

*N* = 225; Nagelkerke R-square = 0.185; Chi-square = 39.636

**p < 0.05

When the other background characteristics of parents and/or care givers were controlled for, as shown in Table 6, the two highest educational categories (post-sec/polytechnic, and university) have statistically significant relationship with the belief that children’s chronic disease can be caused by enemies. Those with Post-secondary education are 84.1% less likely than those with no education to be undecided about the belief that children’s chronic disease can be caused by enemies while those with university education are 94% less likely than those with no education to be undecided about this belief. Some categories of residential status and ethnicity are the only background characteristics that are predictors of care givers’ belief that children’s chronic disease can be caused by enemies when the background characteristics of parents and/or care givers were controlled for. Those with urban residential status are significantly less likely (87.2%) than the rural residents to accept this belief. The Akan and the Ga-Dagme (84% and 96.9%, respectively) are less likely than the Ewe to be undecided about the belief that children’s chronic disease can be caused by enemies.

**Table 6:** Background characteristics and the belief that enemies can cause disease among children

Variables	Yes		Undecided about the belief	
	*P*-values	Odds ratio	*P*-values	Odd ratio
Education				
No education (RC)		1.000		1.000
Primary	0.067	0.221	0.588	1.603
Middle/JSS/JHS	0.061	0.257	0.676	1.434
Sec/SHS/Vocational	0.109	0.278	0.368	0.465
Post-Sec/polytechnic	0.125	0.245	0.029	0.159**
University	0.091	0.222	0.009	0.060**
Sex				
Male	0.070	0.381	0.428	0.672
Female (RC)		1.000		1.000
Religion				
Catholic	0.732	1.410	0.290	0.353
Protestant/Angl/Presb	0.966	1.032	0.526	0.623
Charismatic	0.613	1.445	0.417	0.546
Moslem	0.838	0.788	0.162	0.212
Traditionalist (RC)		1.000		1.000
Residence				
Urban	0.000	0.128**	0.059	0.338
Rural (RC)		1.000		1.000
Ethnicity				
Akan	0.074	0.378	0.001	0.160**
Ga-dagme	0.432	1.751	0.004	0.031**
Other Ghanaians	0.914	1.097	0.726	1.343
Mole-Dagbani	0.471	0.421	0.660	0.626
Ewe (RC)		1.000		1.000
Age group				
< 25 (RC)		1.000		1.000
25–34	0.883	0.915	0.598	0.737
35–44	0.515	1.465	0.268	0.507
45–54	0.460	1.671	0.198	0.354
55–64	0.370	0.372	0.728	1.443
65+	0.911	1.179	0.337	3.675

The reference category is “No”(Does not accept the belief)

Yes = Accepted belief

*N* = 225; Nagelkerke R-square = 0.421; Chi-square = 102.939

**p < 0.05;

#### Education and belief that children suffer from NCDs because of the sins of their parents

Table 7 shows that there is no statistically significant relationship between all the educational categories and the belief that children suffer from NCDs because of the sins of their parents, with the exception of those with Middle/JSS/JHS educational status. The parents and/or care givers with Middle/JSS/JHS are significantly 79.3% less likely than those with no education to accept the belief that NCDs among children are caused by the sins of their parents. Again, they are 92.6% significantly to be undecided about the belief compared with those with no education.

**Table 7:** Education and the belief that children suffer NCDs because of the sins of their parents

Variable	Yes		Undecided about the belief	
	*P*-values	Odds ratio	*P*-values	Odd ratio
Education				
No education (RC)		1.000		1.000
Primary	0.145	0.300	0.052	0.214
Middle/JSS/JHS	0.024	0.207**	0.001	0.074**
Sec/SHS/Vocational	0.253	0.444	0.177	0.423
Post-Sec/Polytechnic	0.156	0.267	0.831	1.143
University	0.081	0.200	0.670	1.286

Note: The reference category is “No” (Does not accept the belief)

Yes = Accepted belief

*N* = 225; Nagelkerke R-square = 0.205; Chi-square = 41.240

**p < 0.05

However, when the background characteristics of parents were controlled for, as shown in Table 8, the Middle/JSS/JHS educational category is the only predictor of whether or not care givers believe that children suffer from NCDs because of the sins of their parents. Compared with those with no education, those with primary and Middle/JSS/JHS education are less likely to be undecided about believing that children suffer NCDs because of the sins of their parents (87.8% and 93.6% respectively). Parents’ sex, religion, place of residence, and ethnicity do not predict this belief.

**Table 8:** Background characteristics and belief that children suffer NCDs because of the sins of their parents

Variables	Yes		Undecided about the belief	
	*P*-values	Odds ratio	*P*-values	Odd ratio
Education				
No education (RC)		1.000		1.000
Primary	0.233	0.332	0.023	0.122**
Middle/JSS/JHS	0.062	0.215	0.003	0.064**
Sec/SHS/Vocational	0.462	0.505	0.922	0.927
Post-Sec/Polytechnic	0.326	0.338	0.447	1.849
University	0.237	0.273	0.148	3.186
Sex				
Male	0.591	1.388	0.085	0.379
Female (RC)		1.000		1.000
Religion				
Catholic	0.450	2.824	0.354	0.387
Protestant/Ang/presby	0.767	1.420	0.662	0.727
Charismatic	0.339	2.959	0.554	0.654
Moslem	0.609	2.252	0.751	0.711
Traditionalist (RC)		1.000		1.000
Residence				
Urban	0.105	0.368	0.234	0.541
Rural (RC)		1.000		1.000
Ethnicity				
Akan	0.664	0.712	0.000	0.053**
Ga-Dagme	0.185	3.672	0.000	0.026**
Other Ghanaians	0.636	1.651	0.116	0.282
Mole-Dagbani	0.398	0.260	0.010	0.046**
Ewe (RC)		1.000		1.000
Age group				
< 25 (RC)		1.000		1.000
25–34	0.077	0.180	0.552	0.712
35–44	0.573	1.507	0.594	0.725
45–54	0.652	1.480	0.159	0.294
55–64	0.416	2.416	0.534	2.053
65+	0.836	1.386	0.854	1.292

The reference category is “No” (Does not accept the belief)

Yes = Accepted belief

*N* = 225; Nagelkerke R-square = 0.441; Chi-square = 100.429

**p < 0.05

#### Education and the belief that someone could be responsible for NCDs among children

Table 9 is on the relationship between level of education and the parents and/or care givers’ belief that someone could be responsible for their children’s NCDs. The results show that there are no significant relationship between education and the belief that someone could be responsible for their children’s illness. When the other background characteristics were controlled for (as shown in Table 10), only a category of the ethnic groups showed a significant relationship with being undecided about this belief, that is whether to accept the belief or reject it. The Ga-Dangme are 85.6% significantly less likely to be undecided about the belief compared with the Ewe.

**Table 9:** Level of education and the belief that someone could be responsible for the children’s illness

Variable	Yes		Undecided about the belief	
	*P*-values	Odds ratio	*P*-values	Odd ratio
Education				
No education (RC)		1.000		1.000
Primary	0.129	0.346	0.064	0.220
Middle/JSS/JHS	0.062	0.318	0.059	0.281
Sec/SHS/Vocational	0.063	0.289	0.205	0.414
Post-Sec/Polytechnic	0.062	0.250	0.556	0.655
University	0.110	0.300	0.439	1.714

The reference category is “No”(Does not accept the belief)

Yes = Accepted belief

*N* = 225; Nagelkerke R-square = 0.132; Chi-square = 28.064

**p < 0.05

**Table 10:** Background characteristics and the belief that someone could be responsible for the children’s illness

Variable	Yes		Undecided about the belief	
	*P*-values	Odds ratio	*P*-values	Odd ratio
Education				
No education (RC)		1.000		1.000
Primary	0.155	0.331	0.057	0.184
Middle/JSS/JHS	0.067	0.272	0.121	0.310
Sec/SHS/Vocational	0.095	0.272	0.382	0.497
Post-Sec/Polytechnic	0.160	0.305	0.765	0.782
University	0.194	0.318	0.409	1.995
*Sex*				
Male	0.063	0.390	0.690	0.840
Female (RC)		1.000		1.000
*Religion*				
Catholic	0.212	0.280	0.364	0.446
Protestant/Angl/Presby	0.550	0.676	0.988	1.011
Charismatic	0.948	0.959	0.880	1.109
Moslem	0.659	0.642	0.349	0.382
Traditionalist (RC)		1.000		1.000
Residence				
Urban	0.076	0.424	0.445	0.679
Rural (RC)		1.000		1.000
Ethnicity				
Akan	0.498	1.455	0.144	0.493
Ga-Dagme	0.507	1.640	0.017	0.144*
Other Ghanaians	0.695	1.388	0.862	1.143
Mole-Dagbani	0.512	2.056	0.434	2.208
Ewe (RC)		1.000		1.000
Age group				
< 25 (RC)		1.000		1.000
25–34	0.349	1.734	0.669	1.255
35–44	0.143	2.357	0.922	1.055
45–54	1.000	1.000	0.075	0.264
55–64	0.465	0.460	0.977	1.027
65+	0.940	1.108	0.906	1.149

The reference category is “No” (Does not accept the belief)

Yes = Accepted belief

*N* = 225; Nagelkerke R-square = 0.281; Chi-square = 64.469

**p < 0.05

## Conclusion

All the educational categories have significant relationship with the belief that enemies can cause NCDs among children. Significant relationship is found between only some educational categories regarding the other types of beliefs- parents’ sin can cause NCDs among children and someone else is responsible for NCDs among children. For example, those with primary school level of education are significantly undecided about belief that enemies can cause NCDs among children compared with those with no education. Also, those with Middle/JSS/JHS education are significantly undecided about the belief that the sin of parents can cause NCDs among children. When the socio-demographic characteristics of parents were controlled for, educational status had a significant relationship with being undecided about the belief that, enemies can cause NCDs among children at the Post-Sec/polytechnic and university levels of education. Being at an urban place of residence also has a significant relationship about the belief that enemies can cause NCDs among children.

Overall, educational status is not always a predictor of whether or not care givers have the belief, reject it or are undecided about the beliefs regarding causes of NCDs among children. With the exception of ethnic background and urban residential status, the other variables are not statistically significant predictors of the beliefs that NCDs among children are caused by enemies, someone else and the sin of parents.

Previous studies about diseases indicate that people have beliefs about some diseases. NCDs are no exception. Generally, Ghanaian society attributes some diseases, particularly NCDs diseases, to the work of spiritual activities and therefore resort to alternative medicine. Consequently, patients arrive at health facilities when the disease has advanced. Deaths from some diseases such as NCDs and even convulsions and malnutrition among children which could be prevented are not because of delay in seeking health care from modern health facilities. This study corroborates findings related to such behaviours [8, 16].

Orthodox medicine may not be the choice for some parents/care givers for the treatment of their children when they have non-communicable diseases as they hold on to such beliefs. They may arrive at the health facility when the illness has reached advanced stage because they would have tried other forms of treatment that are in accordance with the beliefs that they have regarding the cause(s) of the diseases.

Education on the causes of NCDs has to be integrated into the prenatal and post-natal health care delivery system of Ghana to inform parents about the causes of NCDs. At the community level, such education can be done through a number of channels including the media and faith-based institutions. Some of the ethnic groups which have higher likelihood of having these beliefs should be targeted for such educational campaigns.

## Abbreviations

HIV: Human immunodeficiency virus
IMMRI-Noguchi: Memorial medical research institute
IRB: Institutional review board
JHS: Juniour high school
JSS: Juniour secondary school
KATH: Komfo anokye teaching hospital
KBTH: Korle bu teaching hospital
NCDs: Non-communicable diseases
